# Four TRPM4 Cation Channel Mutations Found in Cardiac Conduction Diseases Lead to Altered Protein Stability

**DOI:** 10.3389/fphys.2018.00177

**Published:** 2018-03-08

**Authors:** Beatrice Bianchi, Lijo Cherian Ozhathil, Argelia Medeiros-Domingo, Michael H. Gollob, Hugues Abriel

**Affiliations:** ^1^Swiss National Centre of Competence in Research (NCCR) TransCure, Institute of Biochemistry and Molecular Medicine, University of Bern, Bern, Switzerland; ^2^Department of Cardiology, Inselspital, University Hospital Bern, Bern, Switzerland; ^3^Department of Medicine, Peter Munk Cardiac Centre, Toronto General Hospital, University of Toronto, Toronto, ON, Canada

**Keywords:** TRPM4, protein trafficking, cycloheximide, cardiac disorders, protein stability, ion channels, mutations

## Abstract

Transient receptor potential melastatin member 4 (TRPM4), a non-selective cation channel, mediates cell membrane depolarization in immune response, insulin secretion, neurological disorders, and cancer. Pathological variants in *TRPM4* gene have been linked to several cardiac phenotypes such as complete heart block (CHB), ventricular tachycardia, and Brugada syndrome (BrS). Despite recent findings regarding the functional implications of TRPM4 in cardiac diseases, the molecular and cellular mechanisms leading to altered conduction are poorly understood. In the present study, we identify and characterize four novel *TRPM4* variants found in patients with CHB or ventricular fibrillation. Three of them, p.A101T, p.S1044C and a double variant p.A101T/P1204L, led to a decreased expression and function of the channel. On the contrary, the variant p.Q854R showed an increase in TRPM4 current. Recent evidence indicates that altered degradation rate of mutant proteins represents a pathogenic mechanism underlying genetic diseases. In consequence, protein turnover of WT-TRPM4 and TRPM4 variants overexpressed in HEK293 cells was analyzed using cycloheximide, an inhibitor of protein biosynthesis. Upon addition of cycloheximide, WT-TRPM4 decayed with a half-life of ~20 h, while loss-of-expression variants showed a ~30% increase in degradation rate, with a half-life close to 12 h. Together, the gain-of-expression variant showed a higher stability and a doubled half-life compared to WT-TRPM4. In conclusion, decreased or increased protein expression of several TRPM4 variants linked to cardiac conduction disorders or ventricular arrhythmias were found to be caused by altered TRPM4 half-life compared to the WT form.

## Introduction

The transient receptor potential melastatin 4 (TRPM4) protein is an intracellular Ca^2+^ -activated non-selective cation channel, which is impermeable to Ca^2+^. TRPM4 exhibits a voltage dependence, which has not been previously described for any TRP channel, indicating that TRPM4 is both a Ca^2+^-activated and voltage-modulated cation channel (Nilius et al., 2005). At negative membrane potentials, TRPM4 allows Na^+^ entry into the cell, leading to cellular membrane depolarization. At positive membrane potentials, TRPM4 channels permit cellular K^+^ efflux, leading to membrane repolarization (Launay et al., 2002; Ramsey et al., 2006). TRPM4 is widely expressed in several tissues and is involved in a variety of physiological processes, including modulation of immune cells activity, such as T-cells (Launay et al., 2004), mast cells (Shimizu et al., 2009) and dendritic cells (Barbet et al., 2010), insulin secretion by pancreatic β cells (Cheng et al., 2007), mechano-transduction in cerebral arteries (Vennekens and Nilius, 2007), and Ca^2+^ signaling in cancer (Sozucan et al., 2015; Ceylan et al., 2016; Sagredo et al., 2017). In addition, TRPM4 has been linked to several neurological disorders such as experimental autoimmune encephalomyelitis and multiple sclerosis (Schattling et al., 2012), spinal cord injuries (Gerzanich et al., 2009), and traumatic brain injuries (Simard et al., 2010). TRPM4 was reported to participate in electrical perturbations following cardiac ischemia and reperfusion episodes because of its sensitivity to Ca^2+^ and ATP (Kalogeris et al., 2012). It has been shown that in a model of hypoxia-reoxygenation using isolated mouse interventricular septum, TRPM4 inhibitors 9-phenantrol and flufenamic acid reduced [Ca^2+^]_i_ overload, thus reducing the so called early afterdepolarization (EAD) (Simard et al., 2012). However, the physiological role for this channel in cardiac function remains unclear. Recent studies have linked genetic variants of *TRPM4* gene to progressive familial heart block type 1 (PFHB1) (Kruse et al., 2009; Daumy et al., 2016), isolated cardiac conduction disease (ICCD) (Liu et al., 2010), atrio-ventricular block (AVB) (Stallmeyer et al., 2012; Syam et al., 2016), right bundle branch block (RBBB) (Stallmeyer et al., 2012) and Brugada syndrome (BrS) (Liu et al., 2013; Gualandi et al., 2017). Several TRPM4 variants linked to inherited cardiac diseases were shown to cause either gain- or loss-of-function of the channel activity. However, the molecular details of these alterations, and how both gain- and loss-of-function variants may lead to conduction defects remains poorly understood.

In the present study, we identified four novel *TRPM4* genetic variants found in patients with either complete heart block (CHB) or idiopathic ventricular fibrillation (IVF). Biochemical and functional characterizations of these variants identified three with loss-of-function and one with gain-of-function. We further examined the possible mechanisms leading to altered protein stability using cycloheximide, an inhibitor of protein synthesis. Our results revealed an altered protein turnover in loss- and gain-of-expression mutant compared to the WT form of TRPM4, showing that altered half-life of TRPM4 is one of the mechanisms leading to several cardiac conduction disorders.

## Materials and methods

### Patient genetic screening

The study was conducted according to the Swiss and Canadian guidelines for genetic research and approved by the ethics committees of University Hospital Bern and the University of Ottawa Heart Institute respectively. All subjects gave written informed consent in accordance with the Declaration of Helsinki. Genomic DNA was isolated from peripheral macrophages by standard procedures using Gentra Puregene Blood kit (Qiagen, Hilden, Germany). A targeted exome panel sequencing was performed by Next Generation Sequencing using the TrueSight One Sequencing Panel (Illumina, San Diego, CA, USA). Sequence data were compared with the reference sequence of the human *TRPM4* gene (MIM# 606936; RefSeq NM_017636.3; Ensembl gene reference: ENSG00000130529, referring to isoform *TRPM4b*).

### Generation of TRPM4 mutants

The complete human wild-type HA-tagged *TRPM4* cDNA was cloned in a pcDNA4/TO vector. Mutants were obtained by *in vitro* mutagenesis using Q5 Site-Directed Mutagenesis kit (New England Biolabs, Ipswich, MA, USA). Mutant cDNA clones were then sequenced before using them in experiments.

### Cell culture and transfection

Human embryonic kidney (HEK293) cells were cultured with Dulbecco's modified Eagle's culture medium supplemented with 4 mM Glutamine, 10% FBS and a cocktail of streptomycin-penicillin antibiotics. For the electrophysiological studies, the cells were transiently transfected with 240 ng of HA-TRPM4 WT or HA-TRPM4 p.A101T, HA-TRPM4 p.Q854R, HA-TRPM4 p.S1044C, HA-TRPM4 p.P1204L, and double variant HA-TRPM4 p.A101T/P1204L in a 35 mm dish (BD Falcon, Durham, North Carolina, USA) mixed with 4 μL of JetPEI (Polyplus transfection, Illkirch, France) and 46 μL of 150 mM NaCl. The cells were incubated for 24 h at 37°C with 5% CO_2_. All transfections included 100 ng of eGFP as a reporter gene. Expression of GFP was used to identify transfected cells, for patch clamp experiments.

For the biochemical experiments, HEK293 cells were transiently transfected with 240 ng of either HA-TRPM4 WT, or HA-TRPM4 p.A101T, HA-TRPM4 p.Q854R, HA-TRPM4 p.S1044C, HA-TRPM4 p.P1204L, and double variant HA-TRPM4 p.A101T/P1204L, or empty vector (pcDNA4TO) in a P100 dish (BD Falcon, Durham, North Carolina, USA) mixed with 30 μL of JetPEI (Polyplus transfection, Illkirch, France) and 250 μL of 150 mM NaCl. The cells were incubated for 48 h at 37°C with 5% CO_2_.

### Cell surface biotinylation assay

Following 48 h of incubation, transiently transfected HEK293 cells, previously washed twice with cold 1X PBS, were treated with EZlinkTM Sulfo-NHS-SS-Biotin (Thermo Scientific, Waltham, MA, USA) 0.5 mg/mL in cold 1X PBS for 15 min at 4°C. Subsequently, the cells were washed twice with 200 mM Glycine in cold 1X PBS and twice with cold 1X PBS to inactivate and remove the excess biotin, respectively. The cells were then lysed with 1X lysis buffer [50 mM HEPES pH 7.4; 150 mM NaCl; 1.5 mM MgCl_2_; 1 mM EGTA pH 8.0; 10% Glycerol; 1% Triton X-100; 1X Complete Protease Inhibitor Cocktail (Roche, Mannheim, Germany)] for 1 h at 4°C. Cell lysates were centrifuged at 16,000 g at 4°C for 15 min. Two milligrams of the supernatant were incubated with 50 μL Streptavidin Sepharose High Performance beads (GE Healthcare, Uppsala, Sweden) for 2 h at 4°C, and the remaining supernatant was kept as the input. The beads were subsequently washed five times with 1X lysis buffer before elution with 50 μL of 2X NuPAGE sample buffer (Invitrogen, Carlsbad, CA, USA) plus 100 mM DTT at 37°C for 30 min. These biotinylated fractions were analyzed as TRPM4 expressed at the cell surface. The input fractions, analyzed as total expression of TRPM4, were resuspended with 4X NuPAGE Sample Buffer plus 100 mM DTT to give a concentration of 1 mg/mL and incubated at 37°C for 30 min.

### Western blot experiments

Protein samples were analyzed on 9% polyacrylamide gels, transferred with the TurboBlot dry blot system (Biorad, Hercules, CA, USA) and detected with anti-TRPM4 (generated by Pineda, Berlin, Germany), anti α-actin A2066 (Sigma-Aldrich, St. Louis, MO, USA), anti-alpha Na^+^/K^+^ ATPase α1 ab7671 (Abcam, Cambridge, UK) antibodies using SNAP i.d. (Millipore, Billerica, MA, USA). The anti-TRPM4 antibody was generated by Pineda (Berlin, Germany) using the following peptide sequence: NH2-CRDKRESDSERLKRTSQKV-CONH2. A fraction of the antisera, which was subsequently used in this study, was affinity purified. Actin and alpha-Na^+^/K^+^ ATPase were used to normalize TRPM4 protein band intensity in total fraction and surface fraction respectively. Original Western blot pictures of the three replicates for total and surface fractions were shown in Supplementary Figures 1, 9, and 11 for total expression, and Supplementary Figures 2, 10 and 12 for surface expression. A summary of raw WB picture was also submitted as Supplementary Figure 13. All the Western Blots have been quantified using Image Studio Lite software from LI-COR Biosciences (Lincoln, NE, USA).

### Immunoprecipitation and protein half-life determination experiments

For half-life experiments, HEK 293 transiently transfected cells were treated with 100 μg/mL cycloheximide (Millipore, Billerica, MA, USA) or 0,3% DMSO as control and lysed at various time points using 1X UBI buffer [50 mM HEPES pH 7.4, 150 mM NaCl, 1 mM EGTA pH 8.0, 10% Glycerol, 1% Triton X-100, 1X Complete Protease Inhibitor Cocktail EDTA-free (Roche, Mannheim, Germany)]. Two milligrams of protein lysate were incubated overnight with 20 μg of anti-TRPM4 antibody in a final volume of 1 mL. The lysate-antibody was then transferred on Protein G Sepharose 4 Fast Flow beads (GE Healthcare, Uppsala, Sweden) and rotated overnight at 4°C. Beads were washed 5 times with 1X UBI buffer with 0.5% Triton before elution with 50 μL of 2X NuPAGE sample buffer (Invitrogen, Carlsbad, CA, USA) plus 100 mM DTT at 37°C for 30 min. Samples were loaded on a 9% polyacrylamide gel and TRPM4 expression was revealed using anti-HA antibody (Enzo Life Sciences, Lausen, Switzerland). Original Western blot pictures are shown in Supplementary data (Supplementary Figures 3–8). To extrapolate the half-life of WT-TRPM4 and TRPM4 variants, the relative protein band intensity was fitted with mono-exponential function, and time for 50% decay of intensity was estimated.

### Quantitative real time PCR

Total RNA isolation was performed using TRIzol Reagent (Applied Biosystems, Foster City, CA, USA) as described by the manufacturer. Concentration and purity of total RNA was determined by optical density measurement using a NanoDrop 2000 spectrophotometer (Thermo Scientific, Waltham, MA, USA). cDNA was synthesized using High Capacity cDNA Reverse Transcription kit (Applied Biosystems, Foster City, CA, USA) and quantitative expression analysis was performed with 7500 Fast Real-Time PCR System (Applied Biosystems, Foster City, CA, USA). Quantification of mRNA expression levels was investigated with TaqMan gene expression assay Hs00214167_m1 for human *TRPM4*, using *GAPDH* TaqMan gene expression assay Hs02758991_g1 as a control. Relative expression of the studied gene was calculated with the 2^−ΔΔCt^ method.

### Cellular electrophysiology

Electrophysiological recordings were performed in the inside-out patch clamp configuration with patch pipettes (1–2 μm tip opening) pulled from 1.5 mm borosilicate glass capillaries (Zeitz-Instruments, GmbH, München, Germany) using micropipette puller P 97 (Sutter Instruments, Novato, CA, USA). The tips were polished to have a pipette resistance of 2–4 MΩ in the bath solution. The pipette solution contained (in mM) 150 NaCl, 10 HEPES, and 2 CaCl_2_ (pH 7.4 with NaOH). The bath solution contained (in mM) 150 NaCl 10 HEPES, 2 HEDTA (pH 7.4 with NaOH) as 0 Ca^2+^ solution. Solutions containing 300 μM Ca^2+^ were made by adding the appropriate concentration of CaCl_2_ without buffer to a solution containing (in mM) 150 NaCl, 10 HEPES (pH 7.4 with NaOH) as reported previously (Zhang et al., 2005). Bath solution with 0 and 300 μM Ca^2+^ concentrations were applied to cells by a modified rapid solution exchanger (Perfusion Fast-Step SF-77B; Warner Instruments Corp., CT, USA). Membrane currents were recorded with a Multiclamp 700B amplifier (Molecular Devices, Sunnyvale CA, USA) controlled by Clampex 10 via a Digidata 1332A (Molecular Devices, Sunnyvale, CA, USA). Data were low-pass filtered at 5 kHz and sampled at 10 kHz. Experiments were performed at room temperature (20–25°C). For measuring steady state currents, stimulation protocol consisted of a single 200 ms voltage step to −100 and then to +100 mV.

### Statistical analysis

Data are represented as the mean ± s.e.m. One-way ANOVA followed by Sidak's multiple comparison test was used to compare the samples, with a *p* < 0.05 considered as significant. Electrophysiology data were exported and analyzed using IGOR PRO 6 (Wavematrix, London, UK).

## Results

### Patients information

**Figure 1:**
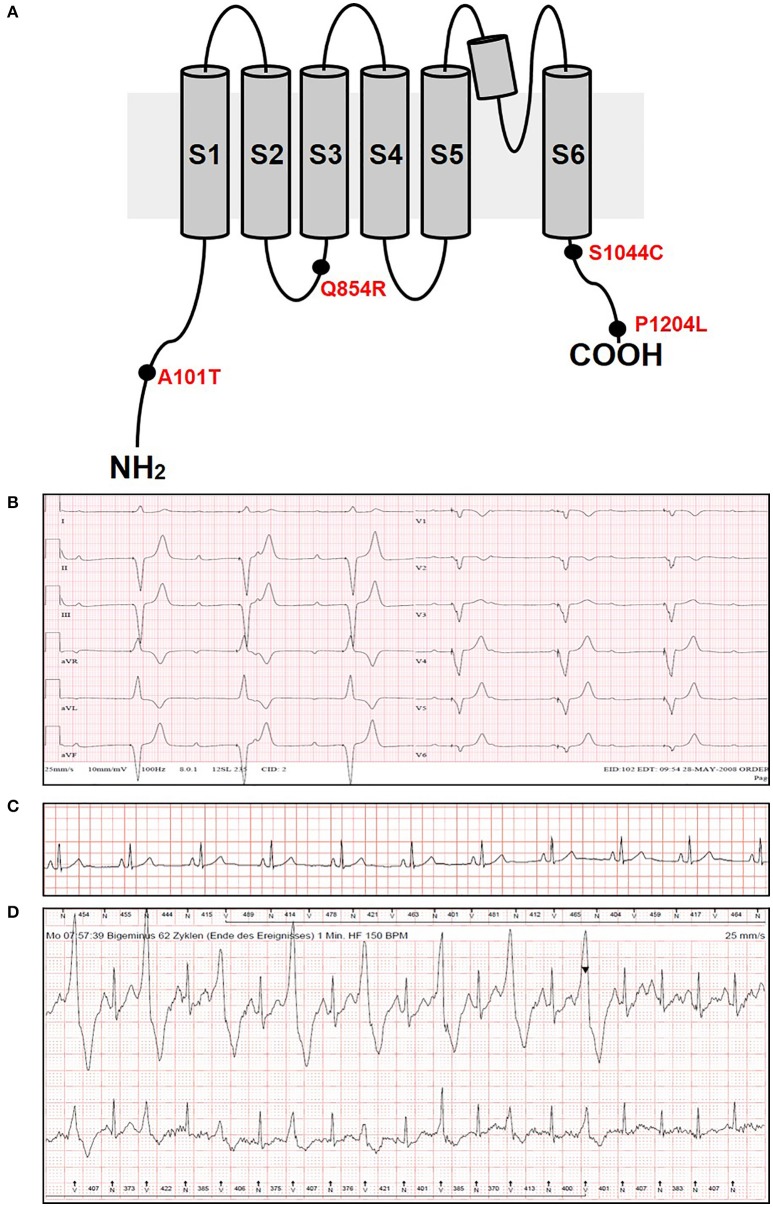
ECG recordings and variants information. **(A)** Variants location in the TRPM4 protein. **(B)** ECG from patient 1 carrying p.A101T variant and showing a complete heart block phenotype. **(C,D)** ECG from patient 5 carrying p.A101T/P1204L variant at rest **(C)** and under physical exercise showing IVF phenotype **(D)**. The arrowhead shows the administration of beta-blockers.

In the present study, we performed genetic analysis of the *TRPM4* gene in patients to elucidate its role in various types of cardiac conduction disorders or ventricular arrhythmias. The clinical details of the variant carriers are presented in Table 1 and the electrocardiogram (ECG) recordings are shown in Figure 1. In Ottawa Heart Institute, 26 patients with premature heart block (age <60 years) were screened. Together, 100 patients from an elderly (age > 70 years) control population were screened for the identified rare variants. In Bern, the *TRPM4* gene is screened routinely in cases with suspected channelopathies. In these cohorts, four novel *TRPM4* variants leading to cardiac disorders were identified (Figure 1A). The p.A101T variant was found in a 54 years old male with dizziness, who was diagnosed with CHB (Figure 1B). The same variant was also found in an asymptomatic control patient who was subsequently identified to have a complete RBBB on ECG. Other variants in the control patient were absent (Table 1). The p.Q854R and p.S1044C variants were found in two female patients with syncope and an ECG presentation of CHB at 53 and 48 years of age respectively. The mother of the 48 years old patient also had a pacemaker (Table 1). The missense p.A101T mutation was also found, together with a p.P1204L mutation, in a 17 years old patient from the University Hospital Bern who had sudden cardiac death. This patient had exhibited frequent monomorphic premature ventricular beats during exercise prior to her sudden cardiac death (SCD) event (Figures 1C,D). She had history of recurrent syncope since she was 14 years old. ECG and magnetic resonance imaging (MRI) were normal. This patient had also a novel homozygous mutation in ryanodine receptor 2 (RYR2) in position c.365G>A (p.R122H), but no clear Catecholaminergic Polymorphic Ventricular Tachycardia (CPVT) was observed. Relatives have been tested for the three mutations: both parents are heterozygous for RYR2-R122H, and the mother is carrier of both TRPM4 mutations. Both parents are asymptomatic and exhibit no obvious cardiac pathological phenotype. Given the uncertainty of the causative genotype, p.P1204L has been also investigated as a single variant, but was already reported to be implicated in cardiac conduction disturbances and/or BrS (Stallmeyer et al., 2012; Liu et al., 2013). To predict the impact of the amino acid substitution, exome variant server (http://evs.gs.washington.edu/EVS/), as well as Polyphen2, SIFT and Mutation Taster were used (Table 2). For data interpretation, human gene mutation database (HGMD, Biobase), 1000 genoms and ExAC browser were used.

**Table 1:** Patients characteristics.

**Patient ID**	**Protein**	**Sex**	**Age at diagnosis (years)**	**Circumstances of discovery**	**Family history**	**Phenotype**
1	p.A101T	Male	54	Dizziness	No	CHB
1A	p.A101T	Male	71	Asymptomatic	No	cRBBB
2	p.Q854R	Female	53	Syncope	Unknown	CHB
3	p.S1044C	Female	48	Syncope	Mother had PM (deceased)	CHB
4	p.A101T/P1204L	Female	17	Resuscitated SCD	Yes	IVF

*CHB, complete heart block; cRBBB, complete right bundle branch block; PM, pacemaker; SCD, sudden cardiac death; IVF, idiopathic ventricular fibrillation*.

**Table 2:** Variants characteristics.

**Patient ID**	**cDNA**	**Exon**	**Protein**	**Predicted protein localization**	**GVS function**	**EVS Eur-Am**	**PolyPhen-2 analysis**
1	c.301G>A	3	p.A101T	N-terminal, cytoplasmic	Missense	A = 161/G = 8439	Benign (Score:0.000)
2	c.2561A>G	17	p.Q854R	Cytoplasmic	Missense	G = 10/A = 8550	Benign (Score:0.008)
3	c.3130A>T	20	p.S1044C	Helical	n.d.	N.P	n.d.
4	c.3611C>T	23	p.P1204L	C-terminal, cytoplasmic	Missense	T = 28/C = 8542	Possibly damaging (Score:0.008)
4	c.301G>A; c.3611C>T	3;23	p.A101T/P1204L	N-terminal, C-terminal, cytoplasmic	n.d.	N.P	n.d.

*EVS Eur-Am, Exome Variant Server European-American Population; GVS, Genome Variation Server; N.P., not present in Exome Variant Server; n.d., not determined*.

### TRPM4 variants expression pattern

**Figure 2:**
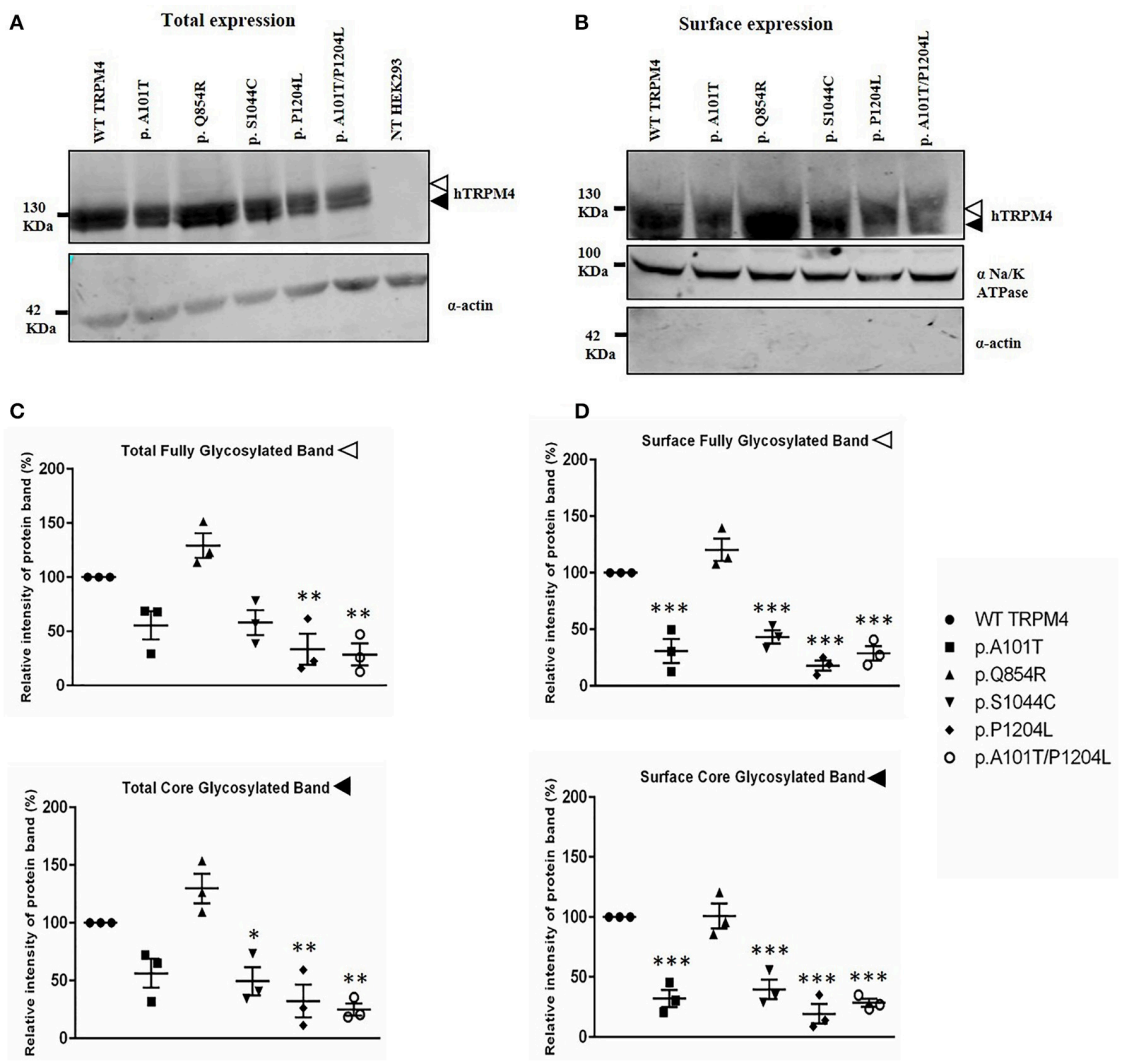
Western Blot analysis of HEK293 cells transiently expressing WT-TRPM4 and TRPM4 variants. Representative immunoblots of human TRPM4 expression at the total **(A)** and surface expression **(B)**. Actin and alpha Na^+^/K^+^ ATPase were used for normalization of the total and the surface expression respectively. White and black arrowheads represent fully and core glycosylated band respectively. Quantification of the total expression **(C)** and surface expression **(D)** is shown as relative intensity of protein band for both full and core glycosylated forms of TRPM4 in each fraction. Data are represented as mean ± s.e.m. of *n* = 3; ^*^P < 0.05; ^**^P < 0.005; ^***^P < 0.001.

**Figure 3:**
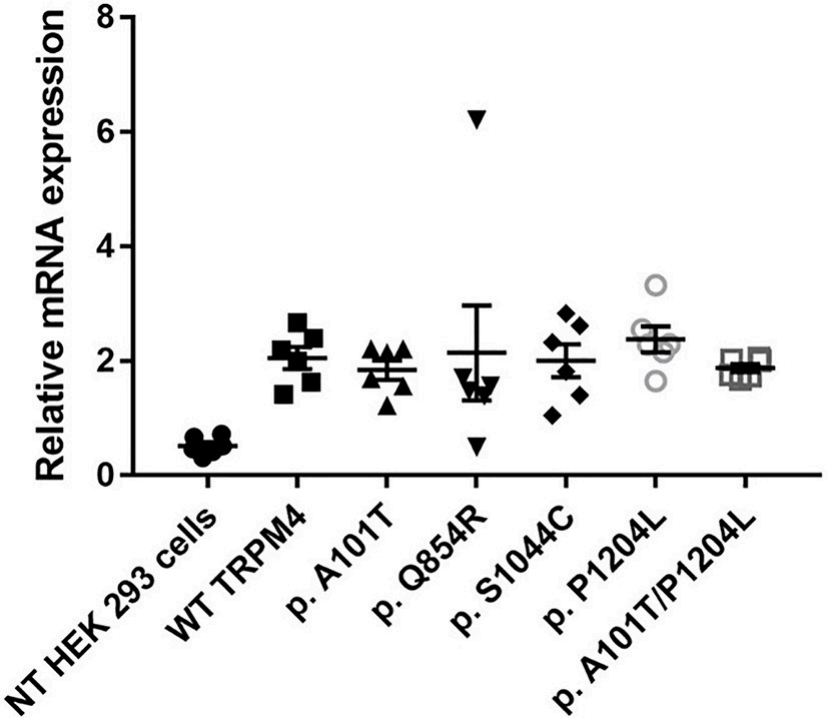
mRNA TRPM4 expression of WT and variants. qPCR analysis on *TRPM4* gene expression in HEK 293 cells transiently transfected with WT-TRPM4 and TRPM4 variants. Data are represented as relative mRNA expression. The expression of *GAPDH* is used as reference (mean ± s.e.m. of *n* = 6). No significant differences are observed between WT and variants.

To understand the consequences of the four novel mutations on the TRPM4 channel, we analyzed total expression of TRPM4 after transient transfection of HEK293 cells and its expression at the cell surface by Western Blot and cell surface biotinylation experiments. As previously reported (Syam et al., 2014), we observed that the TRPM4 channel is expressed in fully and core glycosylated forms. As shown in Figure 2, we observed a 50% reduction in the total expression of p.A101T and p.S1044C, when compared to the WT TRPM4, both in fully and in core glycosylated bands (Figures 2A–C). p.P1204L and the double variant p.A101T/P1204L showed a more severe reduction in the total expression, in both fully and core glycosylated bands (70 and 75% respectively), when compared to WT TRPM4 (Figures 2A–C). The decrease in TRPM4 expression was more pronounced at the cell surface for all the loss-of-expression variants (70% loss for p.A101T and p.A101T/P1204L, 60% loss for p.S1044C, 80% loss for p.P1204L), for both fully and core glycosylated bands (Figures 2B–D). On the other hand, p.Q854R showed an increased protein expression in both fully and core glycosylated bands of total and surface expression (130 and 110% respectively) (Figures 2B–D). However, these increases were not statistically significant. We then performed quantitative Real-Time PCR experiments to determine whether these differences in protein expression may have been due to variable mRNA levels after transient transfection. No significant differences were observed in mRNA expression among the variants when compared to WT *TRPM4* gene (Figure 3).

### Functional analysis

**Figure 4:**
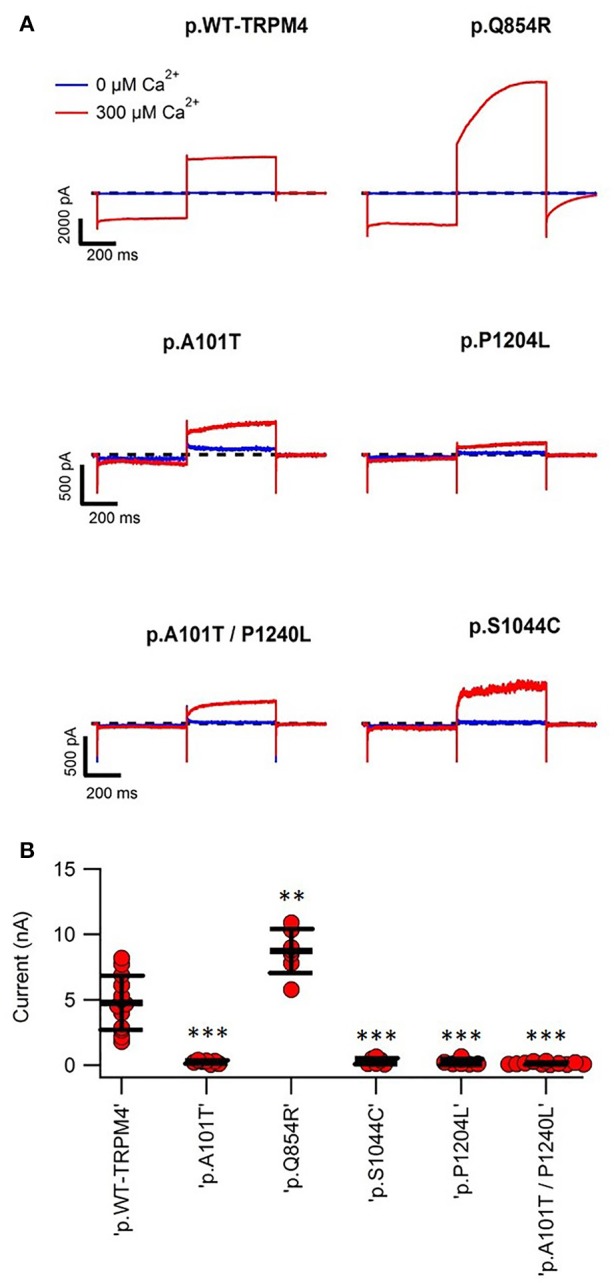
Inside-out patch clamp recordings of WT and TRPM4 variants. **(A)** Individual current traces of WT TRPM4 and TRPM4 variants after applying 300 μM Ca^2+^. **(B)** Quantification of currents of WT and cardiac conduction disorders variants. ^**^P < 0.005; ^***^P < 0.001 (WT, *n* = 13; A101T, *n* = 7; Q854R, *n* = 7; S1044C, *n* = 6; P1204L, *n* = 7; A101T/P1204L, *n* = 11).

To determine possible functional alterations caused by these variants, macro-patch current recordings were performed using inside-out patch clamp on HEK293 cells expressing WT TRPM4 or p.A101T, p.Q854R, p.S1044C, p.P1204L, and p.A101T/P1204L. Figure 4 shows that a 200 ms voltage step from −100 to +100 mV from a holding voltage of 0 mV elicited small outward Na^+^ currents when exposed to nominally 0 Ca^2+^ bath solution (39.6 ± 9.5 pA), whereas it induced large outward currents in presence of 300 μM Ca^2+^ (4768.7 ± 574.6 pA). The amplitude of outward current measured at the end of voltage step to +100 mV varied among the variants. In line with the western blot data (Figure 2), variants p.A101T (249.7 ± 44.5 pA, *n* = 7), p.S1044C (314 ± 95.4 pA, *n* = 6), p.P1204L (248.7 ± 82.9 pA, *n* = 7), and double variant p.A101T/P1204L (142.6 ± 27.7 pA, *n* = 11) showed reduced current while the variant p.Q854R (8735 ± 637.7 pA, *n* = 7) showed increased outward current when compared to WT (4768.7 ± 574.6 pA, *n* = 13; Figure 4B).

### Half-life of WT-TRPM4 and TRPM4 variants

**Figure 5:**
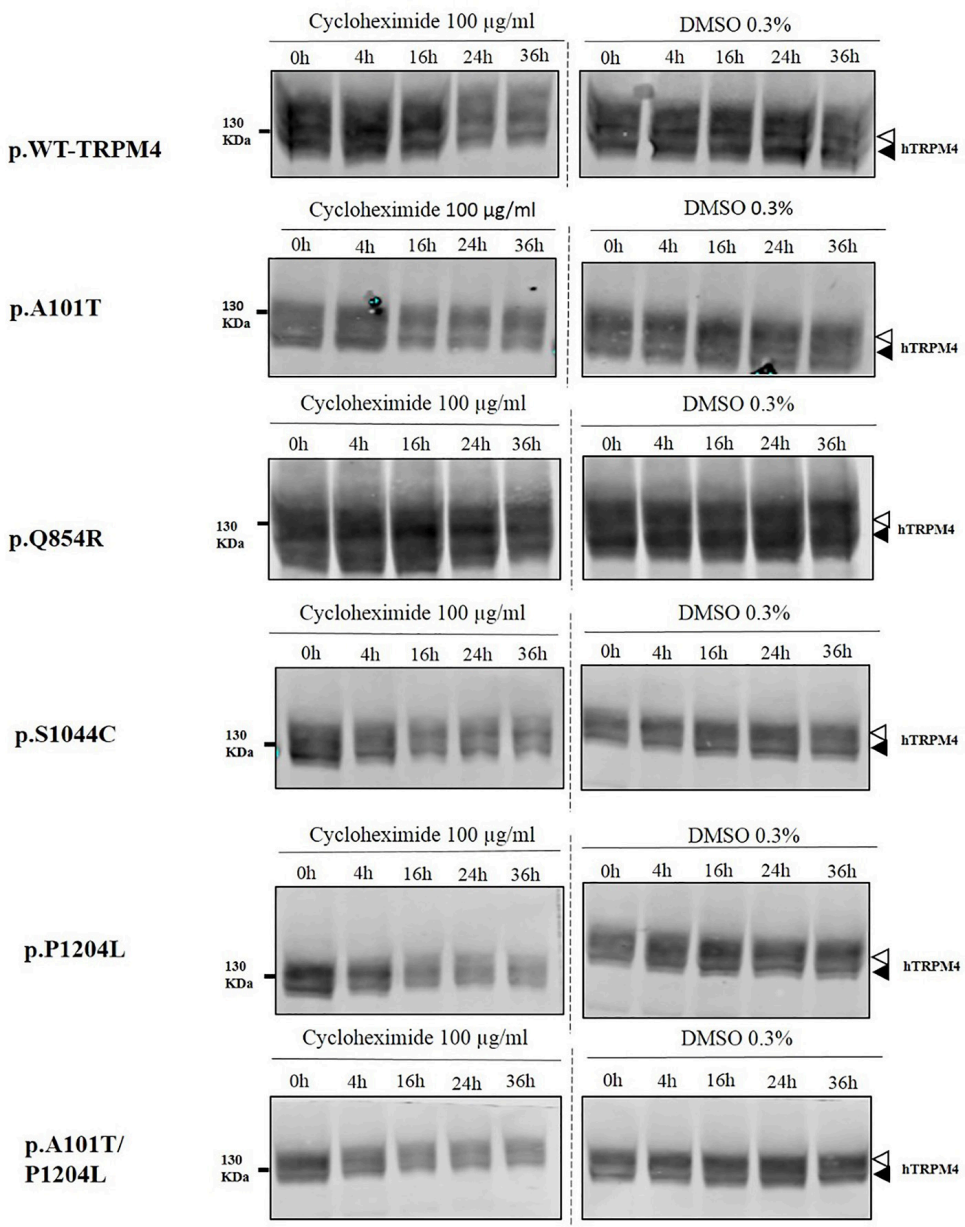
Half-life of WT and TRPM4 variants. Immunoblots showing immunoprecipitation with anti-TRPM4 antibody. Cells were harvested prior to treatment (0 h) and 4, 16, 24, and 36 h after treatment with 100 μg/ml cycloheximide. TRPM4 is detected using anti-HA antibody in presence of 100 μg/ml cycloheximide (left panel), or 0.3% DMSO (right panel). White and black arrowheads represent fully and core glycosylated band respectively. Data are representative of three independent experiments.

**Figure 6:**
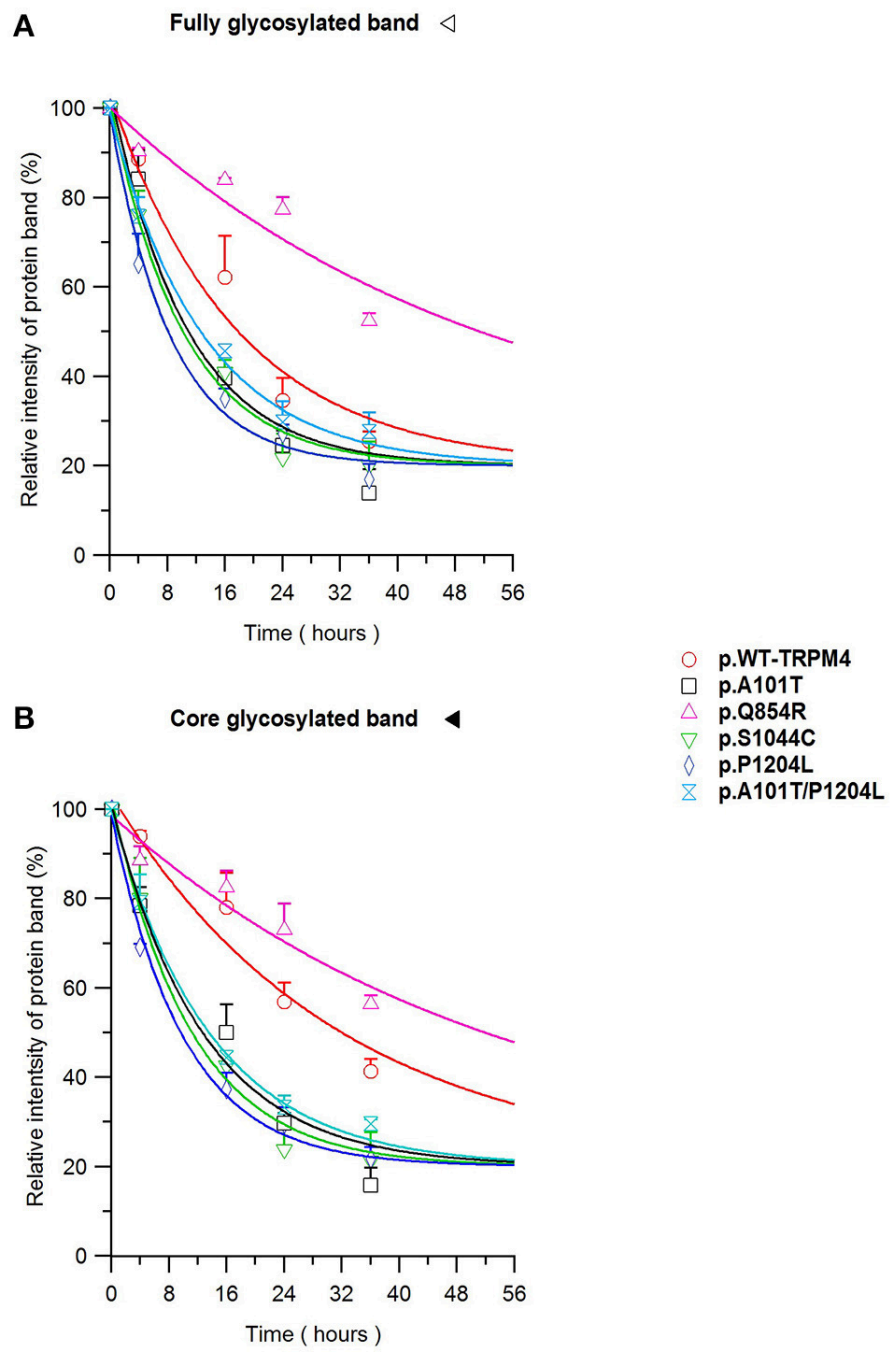
TRPM4 protein expression decay. Immunoblots from Figure 5 are quantified as relative intensity of protein band of the fully **(A)** and the core **(B)** glycosylated band. 0.3% DMSO values are used at each time point for normalization. Statistical analysis has been performed using one-way ANOVA and results are shown in Supplementary Table 1.

To understand the cellular mechanism leading to loss- or gain-of-expression in TRPM4 variants, the half-life of WT and mutated TRPM4 was analyzed using the protein synthesis blocker cycloheximide. Approximately 24 h after transfection, transiently transfected HEK293 cells were incubated with 100 μg/mL cycloheximide and harvested prior to inhibition (0 h) and at 4, 16, 24, and 36 h post-treatment using a TRPM4 immunoprecipitation protocol. For each time point, a 0.3% DMSO control was used to normalize TRPM4 expression. The observed pattern of WT-TRPM4 protein expressed over time following cycloheximide treatment (Figure 5) provided a half-life of ~18 h for the fully glycosylated form, and 30 h for the core glycosylated form (Figure 6 and Table 3). Interestingly, in presence of cycloheximide we observed a more rapid decay in TRPM4 expression of all loss-of-expression variants, with a half-life of ~12 h for p.A101T, p.S1044C and the double variant p.A101T/P1204L, for both fully and core glycosylated fractions (Figures 5, 6 and Table 3). p.P1204L was even more rapidly degraded, and the signal was only partially detectable after 10 h incubation, leading to a half-life of both fully and core glycosylated band of ~8 h (Figure 6 and Table 3). Under the same conditions, gain-of-expression p.Q854R variant half-life has been estimated, resulting to exceed 24 h, and reaching 50 h in both fully and core glycosylated band (Figure 6 and Table 3). Taken together, these experiments indicate that the difference in plasma membrane expression of WT-TRPM4 and TRPM4 variants reflects different degradation kinetics, which causes variations in protein expression.

**Table 3:** Variants half-life values.

**Variant**	**Fully glycosylated band half-life values (hours ± s.e.m.)**	**Core glycosylated band half-life values (hours ± s.e.m.)**
WT-TRPM4	17.4 ± 1.6	31.4 ± 2.6
p.A101T	10.5 ± 1.1	12.7 ± 1.2
p.Q854R	52.3 ± 6.8	53.8 ± 4.7
p.S1044C	10.1 ± 0.7	11.0 ± 0.6
p.P1204L	8.3 ± 0.6	9.9 ± 0.4
p.A101T/P1204L	13.0 ± 0.6	13.8 ± 0.4

## Discussion

In the present study, four novel *TRPM4* variants have been identified in patients with CHB and IVF. Biochemical and electrophysiological studies revealed a reduced protein expression and function for p.A101T, p.S1044C, and p.A101T/P1204L compared to WT, while p.Q854R showed an increased TRPM4-mediated current. Furthermore, we observed that, when expressed in HEK293 cells, these variant TRPM4 proteins had either increased or decreased degradation rates when compared to WT protein.

The underlying mechanisms leading to conduction defects caused by TRPM4 mutations are not yet understood. Moreover, in this work, both gain and loss of function variants led to the same pathology. A similar observation was made in a series of Brugada cases with mutations in the TRPM4 channel (Liu et al., 2013) and in another series of patients with congenital or childhood atrioventricular block (Syam et al., 2016). While it may seem paradoxical that both gain- and loss-of-function can lead to similar phenotype, one may propose that, if TRPM4 is influencing the resting membrane potential of conduction system cells, both membrane hyperpolarization and depolarization will cause conduction slowing (Abriel et al., 2012), in analogy to the supernormal conduction phenomenon which has been described in atrioventricular conduction (Moore et al., 1993). However, to date, there is no report demonstrating a direct role of TRPM4 in this phenomenon, hence motivating further investigation on the role of TRPM4 in the conduction pathway (Mathar et al., 2014). The presence of a TRPM4 loss-of-function variant in a case with IVF is intriguing and we can thus far only speculate that its possible consequences on impulse propagation may exacerbate the arrhythmogenic potential of the observed RYR2 mutation. It is however impossible to exclude that this presents a variant with no direct functional consequences *in vivo*. A recent report investigated the involvement of TRPM4 in conduction block and arrhythmic phenomena associated with cardiac remodeling and injury, showing that TRPM4 channel is activated by a physiological range of Ca^2+^ concentrations and that its excessive activity can cause arrhythmic changes (Hu et al., 2017). In mouse ventricles, a negative impact of TRPM4 channel activity on cardiac contractility despite action potential prolongation has been proposed, and this mechanism was found to be suppressed in TRPM4 KO mice (Mathar et al., 2014). Further improvement of action potential models may help to understand more complex mechanisms underlying arrhythmias and conduction failures associated with TRPM4 genetic mutations.

Using cycloheximide, we investigated whether the variant-induced altered expression of TRPM4 in HEK293 cells may result from modified protein stability. TRPM4 half-lives of the core and fully glycosylated fractions have been determined for the WT and variant proteins causing conduction defects. Our findings revealed that the fully and the core glycosylated fractions of WT channels had half-lives of ~20 and ~30 h, respectively. These values are in line with Woo and colleagues' study, where they observed a half-life of ~24 h for WT mouse TRPM4 (Woo et al., 2013). Together, loss-of-expression mutants undergo a more rapid degradation rate, resulting in reduced half-life compared to WT. Interestingly, the mutation leading to gain-of-expression significantly decreased the degradation rate of the fully and core glycosylated fractions, with a half-life of ~50 h. In contrast, the loss-of-expression variant half-life alterations were mainly seen in the core glycosylated fractions. These observation are consistent with a model where the decrease and increase of TRPM4 currents are due to changes in TRPM4 channel densities at the plasma membrane caused by variant-induced trafficking alterations. However, it is likely that the mechanisms leading to the loss of expression are not the same as the one underlying the gain of expression. It can be hypothesized that the increased degradation rates of the core-glycosylated fractions reflect misfolding of the nascent proteins and subsequent ER-associated protein degradation (ERAD) (Meusser et al., 2005). In a recent study (Syam et al., 2016), two loss-of-expression TRPM4 variants found in patients with congenital AVB were rescued upon decreased incubation temperature for 24 h suggesting that variant-induced misfolding of TRPM4 may be a common molecular mechanism similar to the case of human ether-a-go-go-related gene voltage-gated potassium channel Kv11.1 (Zhou et al., 1999). On the other hand, assuming that the fully glycosylated TRPM4 fraction is predominant at the plasma membrane, it can be speculated that the reduced degradation of the p.Q854R variant reflects a decreased internalization rate as proposed for other gain of function variants (Kruse et al., 2009). Future experiments will have to be performed to assess this hypothesis.

Ion channels, including many TRP channels, are subjected to post-translational modifications which regulate their trafficking and stability at the cell membrane. It has been reported that human and mouse TRPM4 are N-linked glycosylated at a unique residue, Asn^992^ and Asn^988^ respectively (Woo et al., 2013; Syam et al., 2014). N-glycosylation has been found to be crucial for surface expression of several TRP channels, but most studies have been performed on heterologous systems, with induced ion channels overexpression; therefore, native and recombinant proteins may act differently due to differences in *in vivo* glycosylation. Recent reports suggested the role of SUMOylation as a possible post-translational modification implicated in TRPM4 trafficking. Kruse and colleagues proposed that the p.E7K TRPM4 mutation leading to a gain-of-function and expression was subjected to an increased SUMOylation which augments its membrane expression (Kruse et al., 2009). In contrast, Syam et al. showed immunoprecipitation experiments with WT TRPM4 and the gain-of-expression variant p.G582S, where no direct SUMOylation of TRPM4 seemed to be observed (Syam et al., 2016). Ubiquitination is another frequent post-translational modification of ion channels. In our previous work (Syam et al., 2016), we performed GST-S5a pull down experiments to address whether ubiquitination was involved in differences in expression and degradation between WT and variants. Our findings revealed that the difference in degradation rate of WT and mutants does not depend on a different rate of ubiquitination. Based on these contrasting results, the involvement of SUMOylation and/or ubiquitination leading to increased TRPM4 expression in patients with cardiac conduction disorders still remains to be elucidated.

Recently, the electron cryo-microscopy structure of the human TRPM4 has been reported (Winkler et al., 2017). It is shown there that TRPM4 is composed by four cytosolic C-terminal domains which form an umbrella-like structure, with a coil domain for the “pole” and four N-terminal helical “ribs.” p.A101T and p.P1204L are found in well conserved N-terminal and C-terminal residues respectively, while p.Q854R is located in the S2-S3 linker and does not seem to play a crucial role in TRPM4 structure. Interestingly, p.S1044C has been identified to be located in the intracellular gate and might play a role in TRPM4 permeability to Na^+^ (Winkler et al., 2017). Further studies would be required to assess S1044 specific role in the channel function.

## Limitations of the study

The novel TRPM4 variants described in this work were not assessed in terms of their intrinsic biophysical properties. Further studies using cardiac cells instead of HEK 293 cells may give additional information regarding their relevance in action potential conduction and could lead to a better understanding of the role of TRPM4 in cardiac conduction disorders. In conclusion, although the molecular mechanism underlying protein degradation dysfunctions and their role in cardiac diseases are only beginning to be revealed, it has become clear that altered protein degradation affecting protein half-life may play an important role in a variety of genetic cardiac disorders, including cardiac conduction defects in presence of TRPM4 mutations. Further studies, using knock-in mice or CRISPR/Cas9-dependent editing of human iPS-derived cardiomyocytes, may help to develop clinically relevant strategies to correct protein trafficking abnormalities, including misfolded TRPM4 mutants in cardiac conduction phenotypes.

## Author contributions

BB and HA: conceived the study and wrote the manuscript; LO and BB: contributed to the biochemical and functional analyses; MG and AM-D: recruited the patients and provided expert clinical advice. All authors interpreted the data, contributed and commented on drafts of the article, and approved the final version.

### Conflict of interest statement

The authors declare that the research was conducted in the absence of any commercial or financial relationships that could be construed as a potential conflict of interest.

## Supplementary material

Supplementary Figure 1 Raw WB picture 1 of total expression.

Supplementary Figure 2 Raw WB picture 1 of surface expression.

Supplementary Figure 3 Raw WB picture of WT-TRPM4 half-life.

Supplementary Figure 4 Raw WB picture of p.A101T half-life.

Supplementary Figure 5 Raw WB picture of p.Q854R half-life.

Supplementary Figure 6 Raw WB picture of p.S1044C half-life.

Supplementary Figure 7 Raw WB picture of p.P1204L half-life.

Supplementary Figure 8 Raw WB picture of p.A101T/P1204L half-life

Supplementary Figure 9 Raw WB picture 2 of total expression.

Supplementary Figure 10 Raw WB picture 2 of surface expression.

Supplementary Figure 11 Raw WB picture 3 of total expression.

Supplementary Figure 12 Raw WB picture 3 of surface expression.

Supplementary Figure 13 Summary of raw WB used for Figures 2A,B.

Supplementary Table 1 Half-lives statistical values.
