# Supplementary figures and images for: Four TRPM4 Cation Channel Mutations Found in Cardiac Conduction Diseases Lead to Altered Protein Stability
Source: Front Physiol. 2018 Mar 8;9:177. doi: 10.3389/fphys.2018.00177 (PMC5852105; doi:10.3389/fphys.2018.00177)

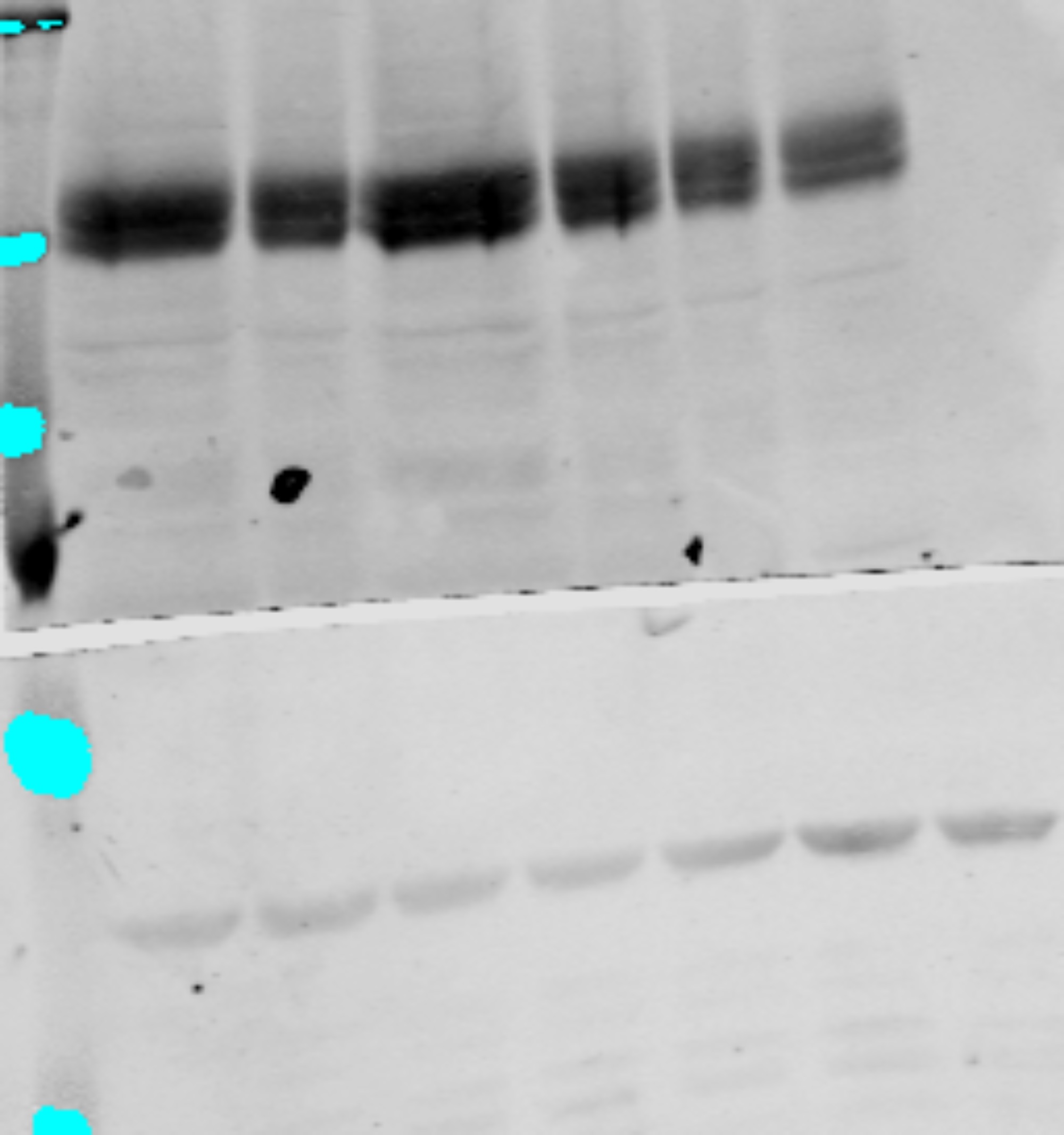

Supplement: Supplementary Figure 1 — Raw WB picture 1 of total expression. [file Image1.JPEG]

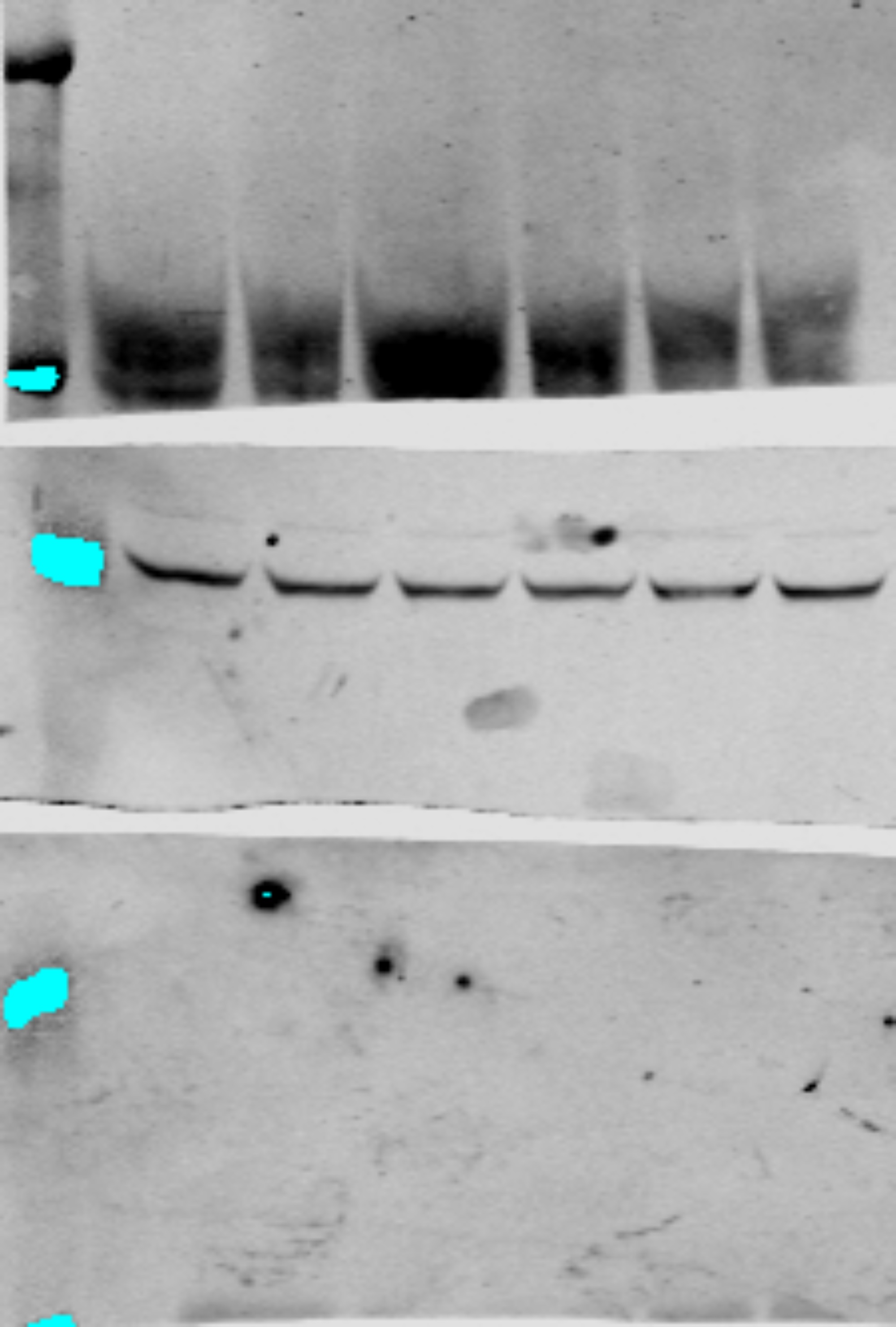

Supplement: Supplementary Figure 2 — Raw WB picture 1 of surface expression. [file Image2.JPEG]

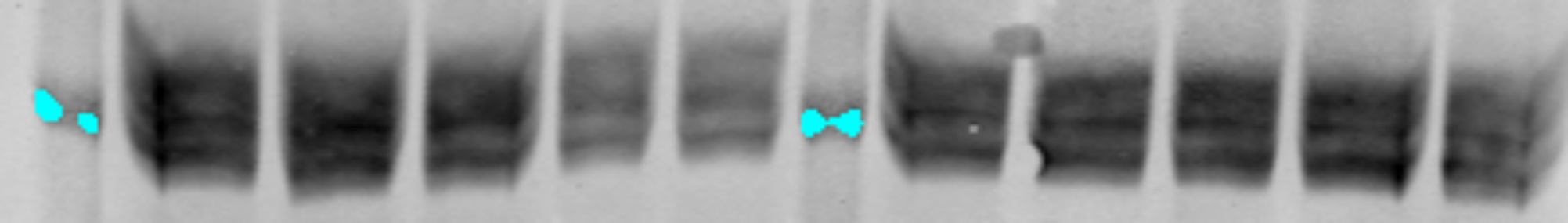

Supplement: Supplementary Figure 3 — Raw WB picture of WT-TRPM4 half-life. [file Image3.JPEG]

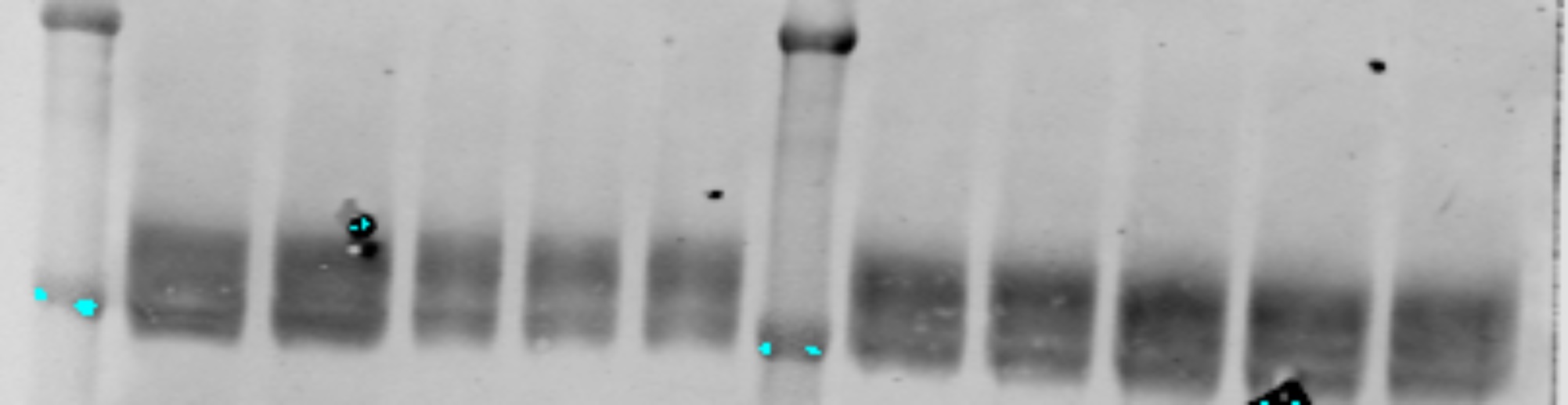

Supplement: Supplementary Figure 4 — Raw WB picture of p.A101T half-life. [file Image4.JPEG]

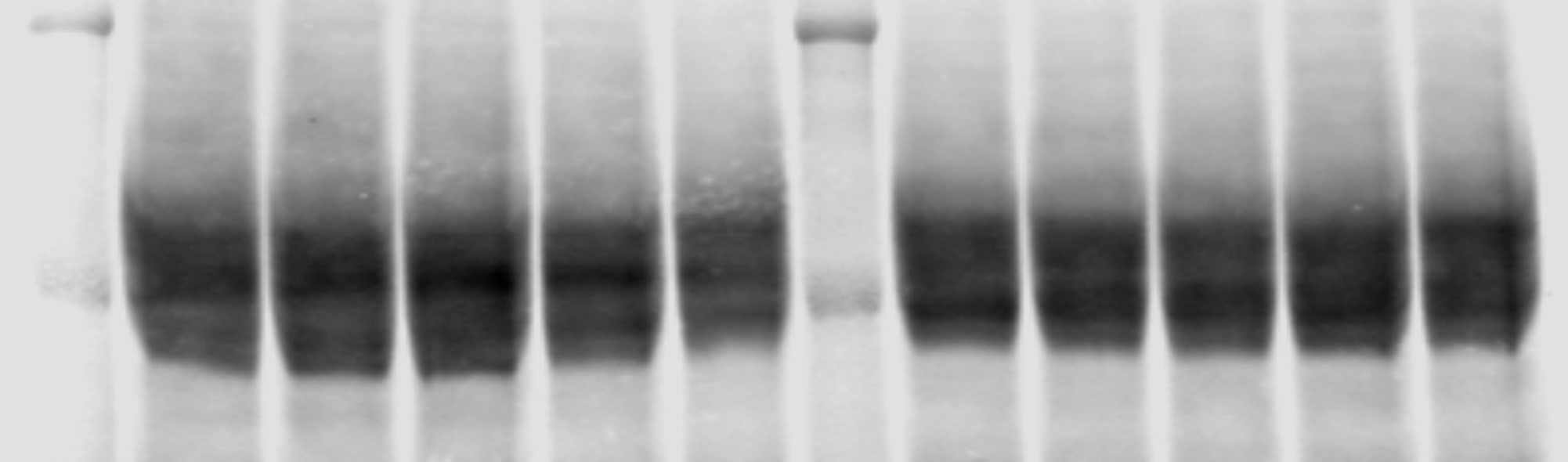

Supplement: Supplementary Figure 5 — Raw WB picture of p.Q854R half-life. [file Image5.JPEG]

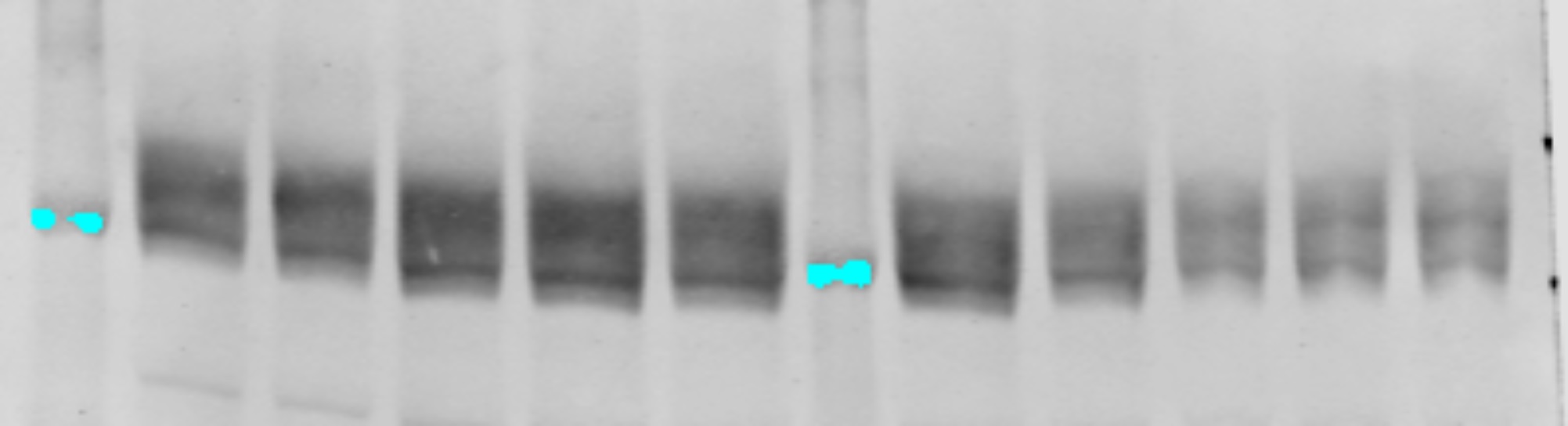

Supplement: Supplementary Figure 6 — Raw WB picture of p.S1044C half-life. [file Image6.JPEG]

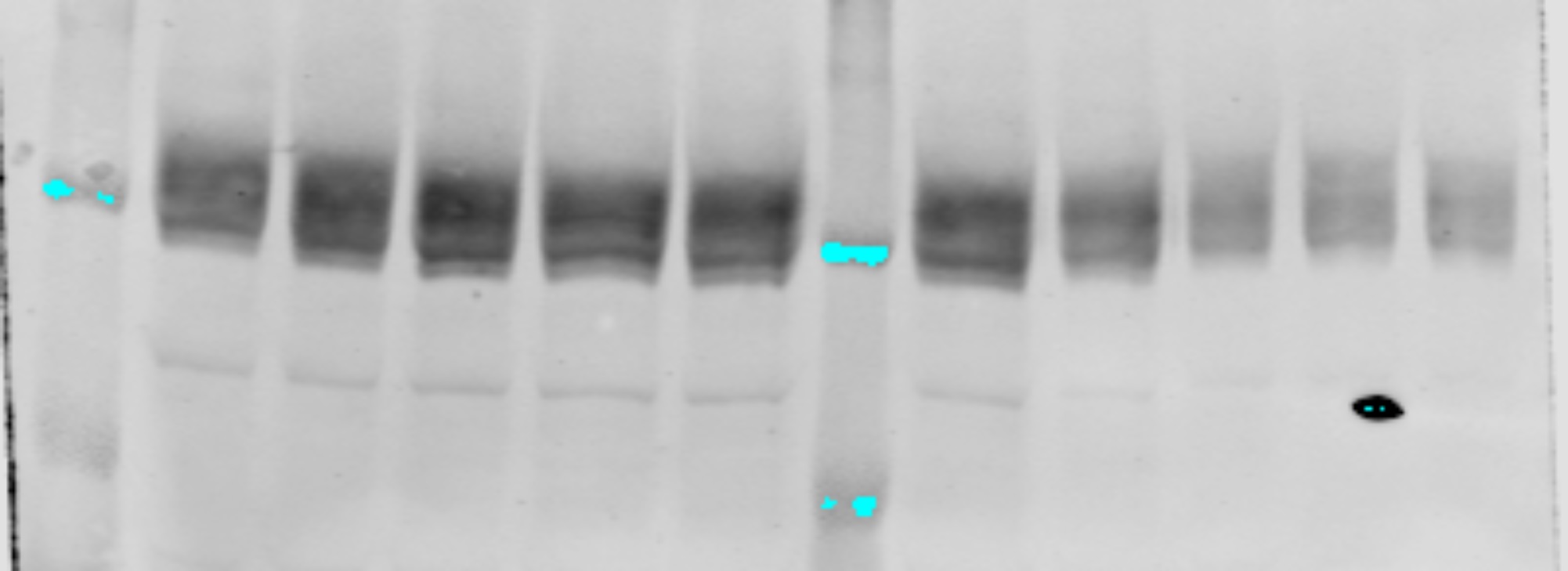

Supplement: Supplementary Figure 7 — Raw WB picture of p.P1204L half-life. [file Image7.JPEG]

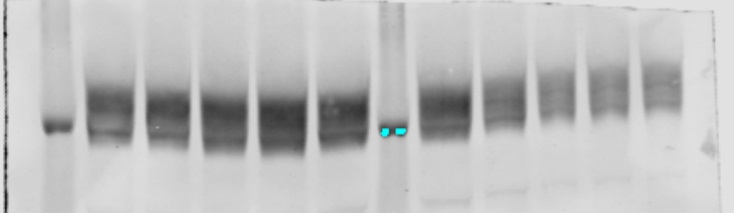

Supplement: Supplementary Figure 8 — Raw WB picture of p.A101T/P1204L half-life [file Image8.JPEG]

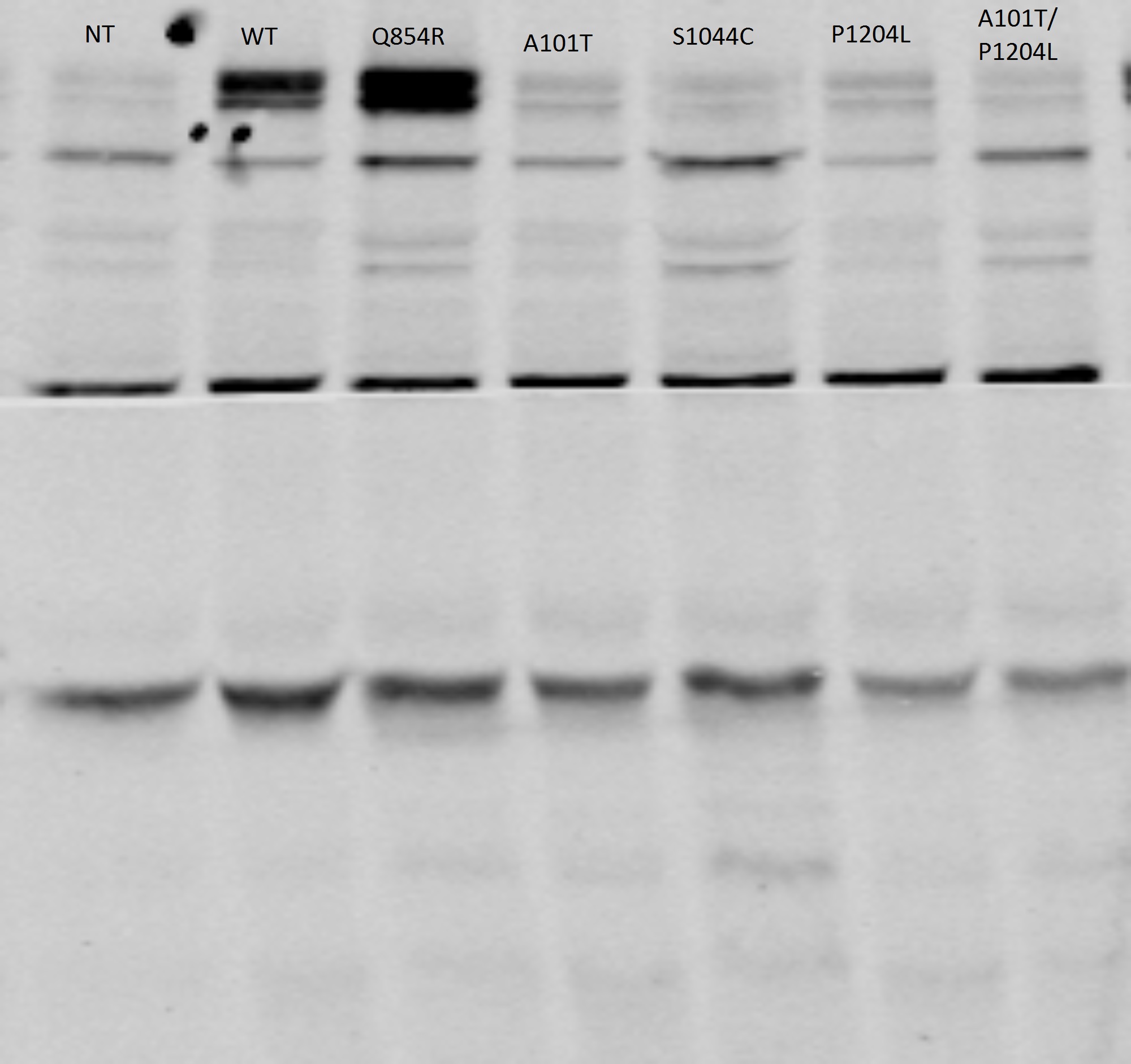

Supplement: Supplementary Figure 9 — Raw WB picture 2 of total expression. [file Image9.JPEG]

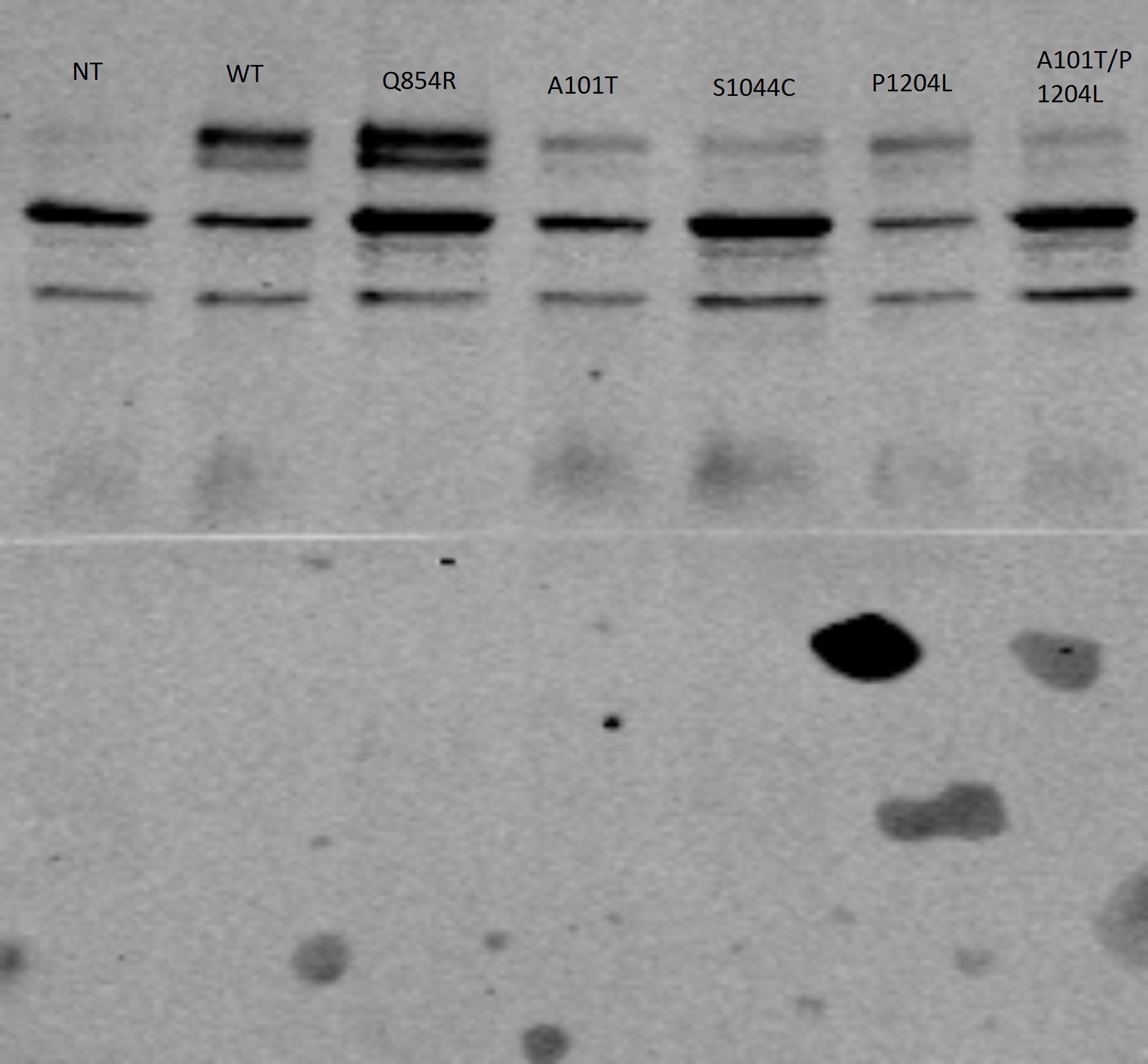

Supplement: Supplementary Figure 10 — Raw WB picture 2 of surface expression. [file Image10.JPEG]

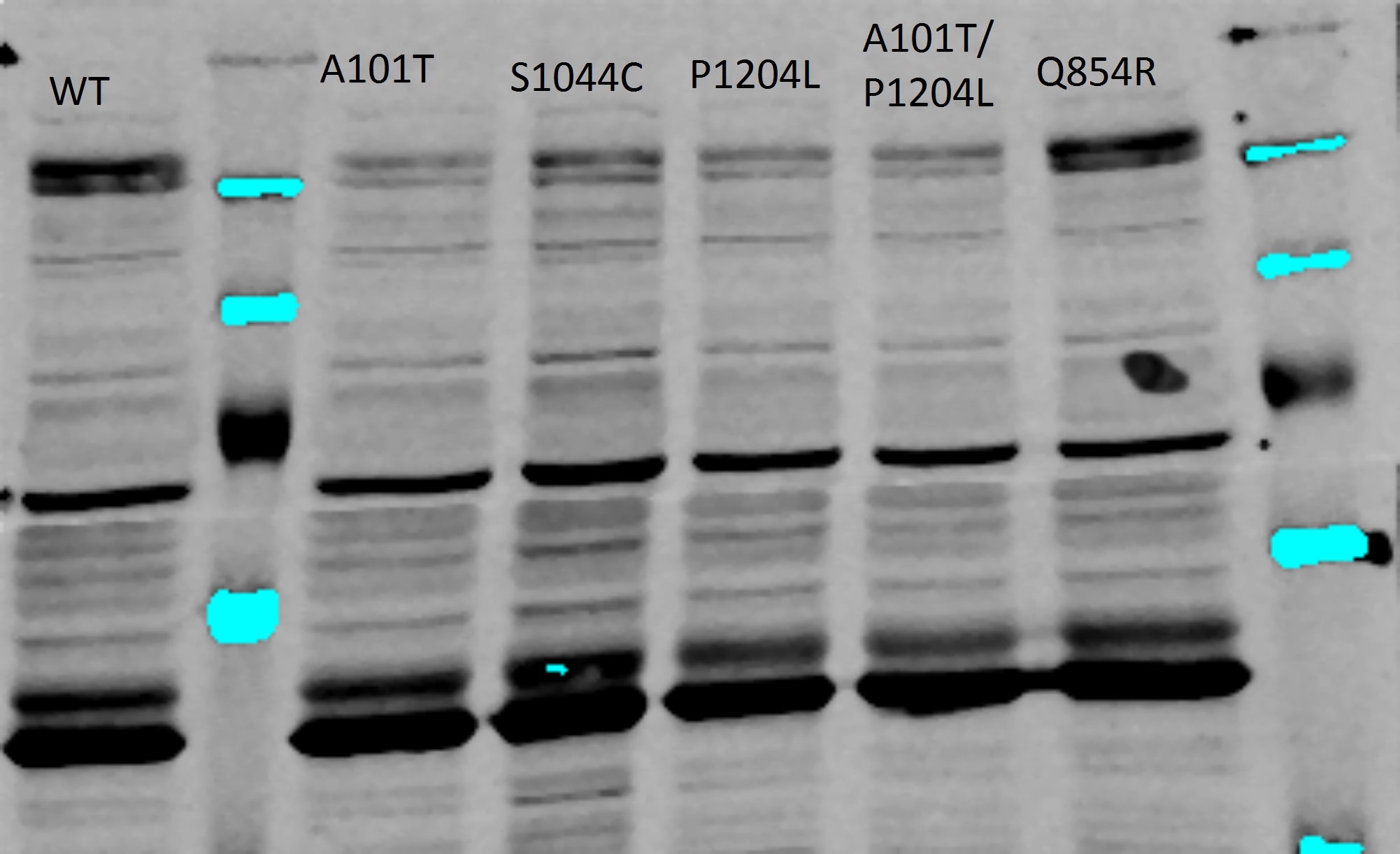

Supplement: Supplementary Figure 11 — Raw WB picture 3 of total expression. [file Image11.JPEG]

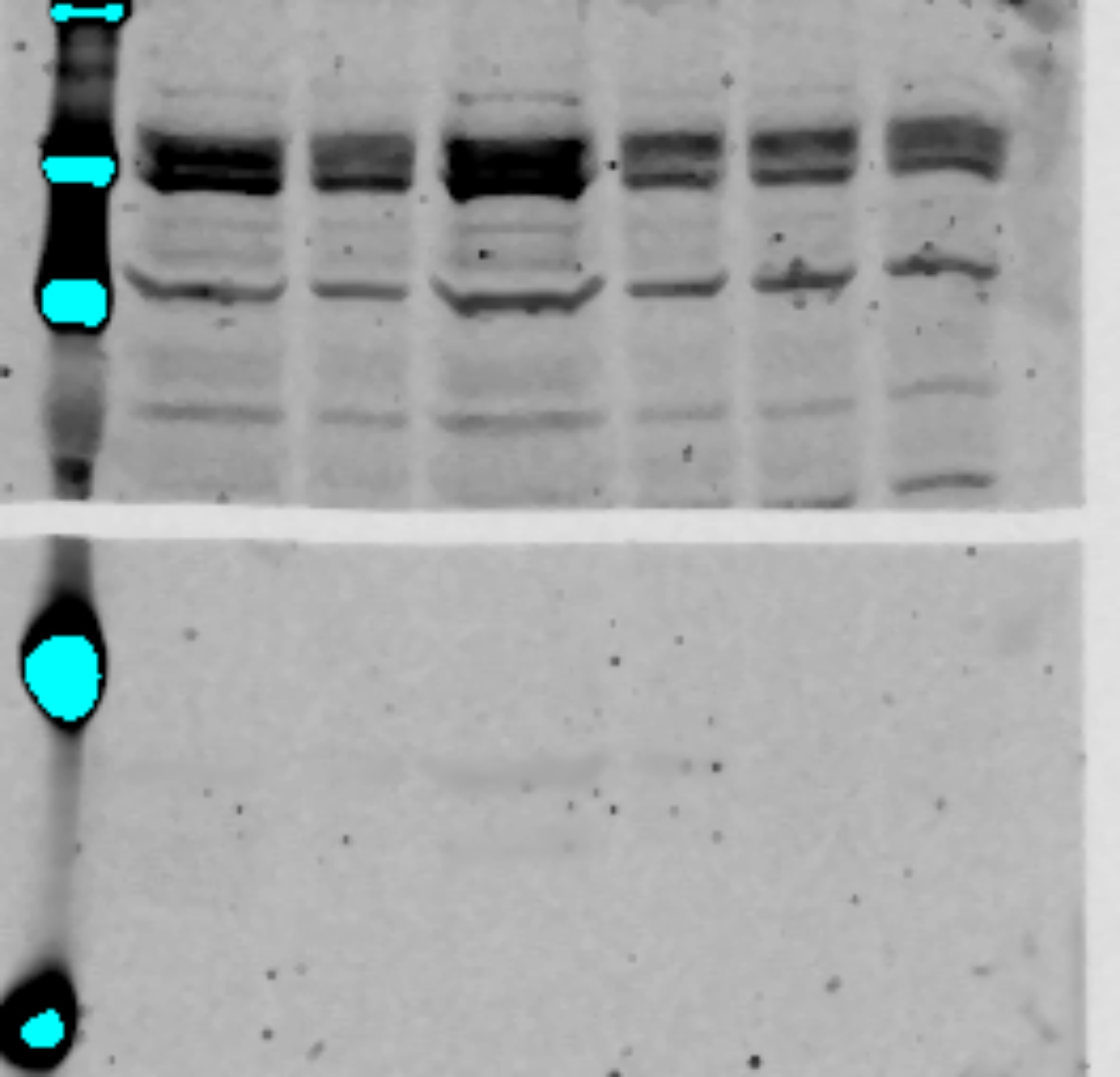

Supplement: Supplementary Figure 12 — Raw WB picture 3 of surface expression. [file Image12.JPEG]

Total expression (Figure 2A)

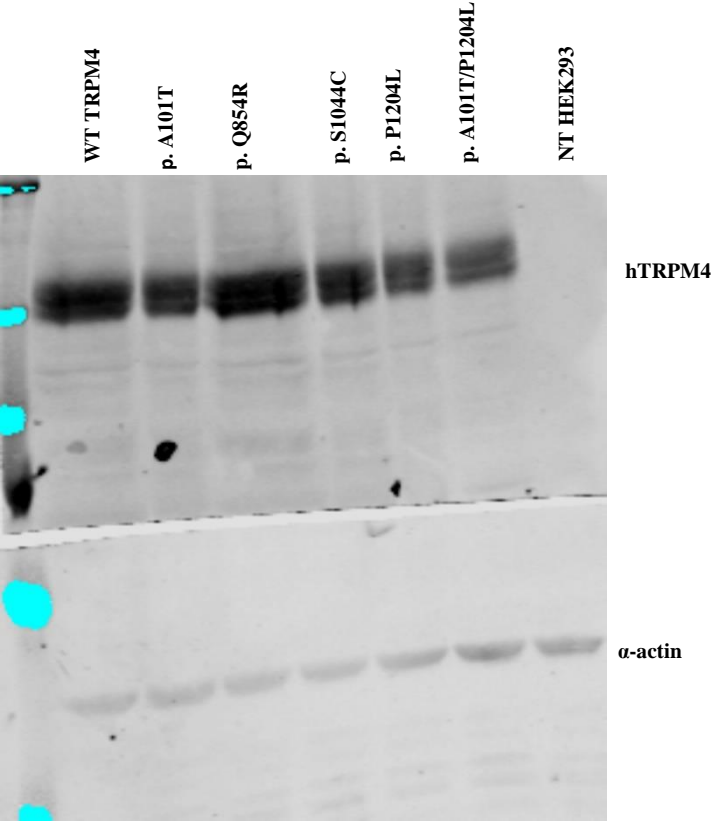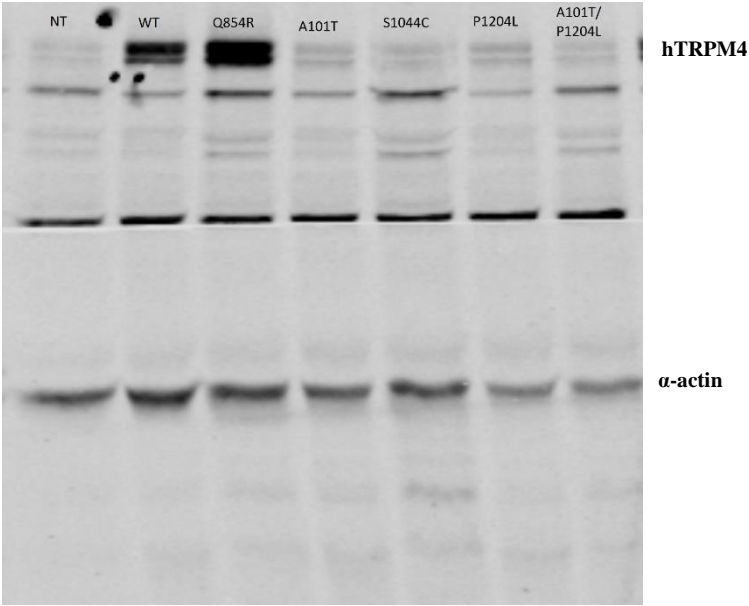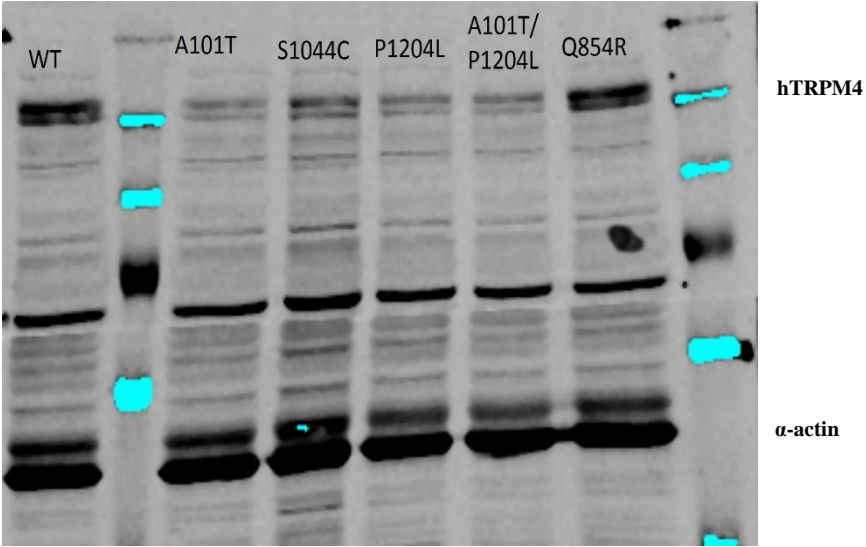

Surface expression (Figure 2B)

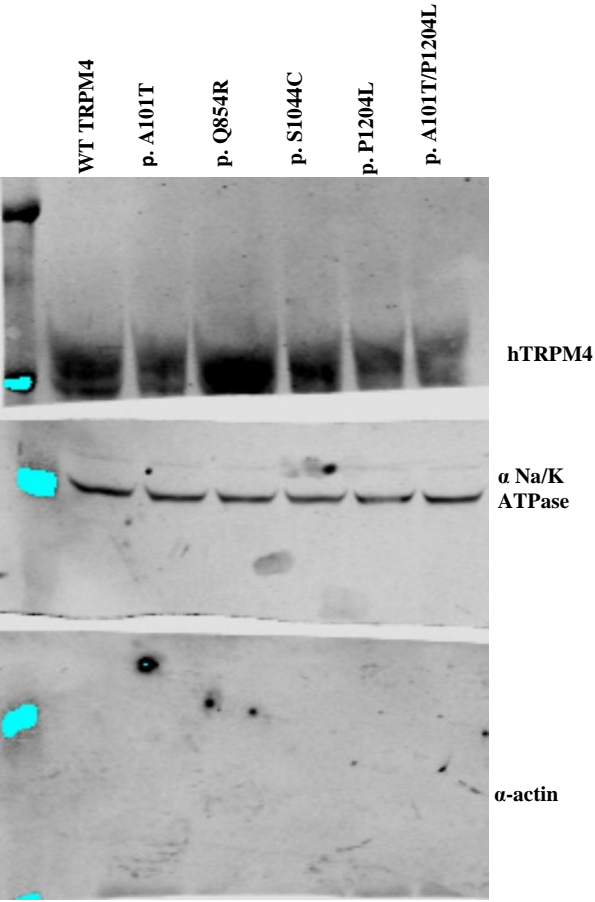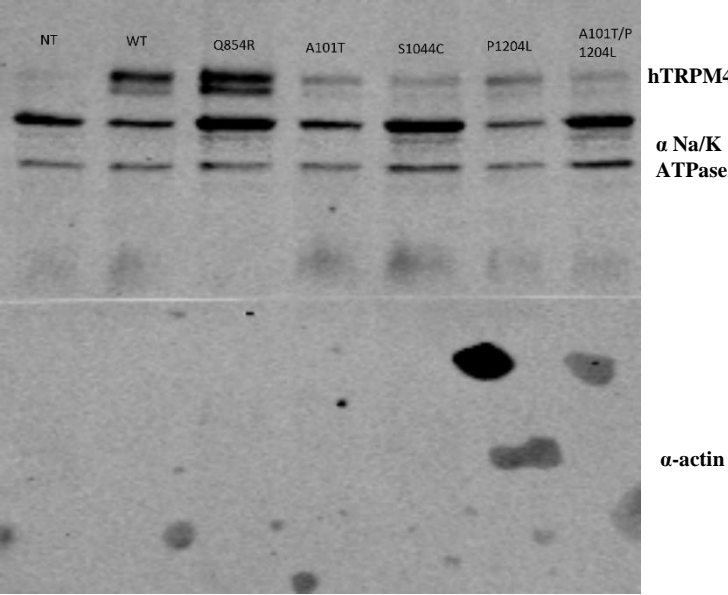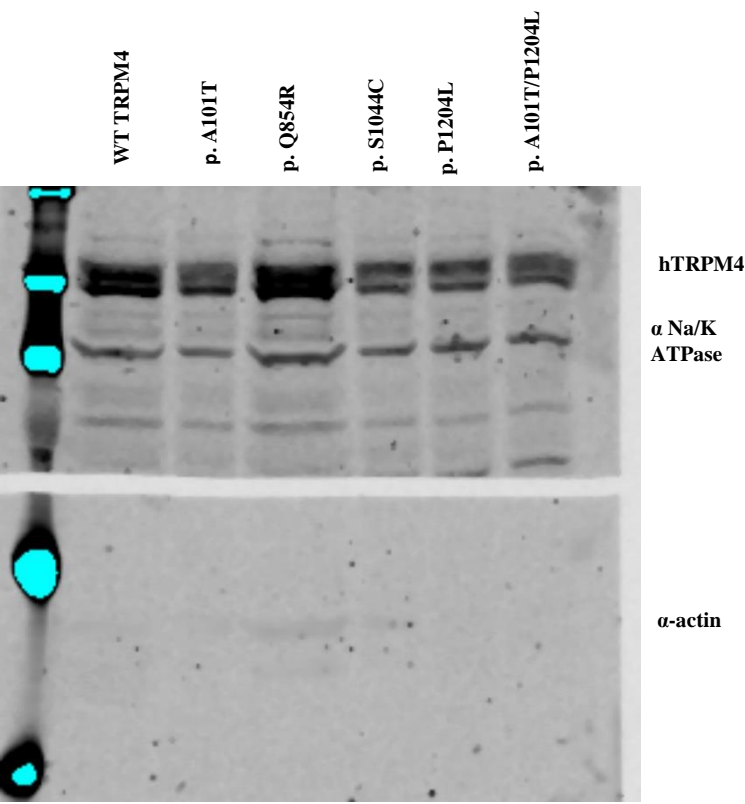

Supplement: Supplementary Figure 13 — Summary of raw WB used for Figures 2A,B. [file Image13.PDF]
