# Supplementary material for: Four TRPM4 Cation Channel Mutations Found in Cardiac Conduction Diseases Lead to Altered Protein Stability
Source: Front Physiol. 2018 Mar 8;9:177. doi: 10.3389/fphys.2018.00177 (PMC5852105; doi:10.3389/fphys.2018.00177)
Supplement: Supplementary Table 1 — Half-lives statistical values. [file Table1.PDF]

Supplementary Table 1 Half-lives statistical values

FULLY GLYCOSYLATED BAND

	4h	16h	24h	36h
**WT-TRPM4 vs p.A101T**	ns	*	ns	ns
**WT-TRPM4 vs p.Q854R**	ns	*	***	**
**WT-TRPM4 vs p.S1044C**	ns	*	ns	ns
**WT-TRPM4 vs p.P1204L**	*	**	ns	ns
**WT-TRPM4 vs p.A101T/P1204L**	ns	ns	ns	ns

CORE GLYCOSYLATED BAND

	4h	16h	24h	36h
**WT-TRPM4 vs p.A101T**	ns	**	**	**
**WT-TRPM4 vs p.Q854R**	ns	ns	ns	ns
**WT-TRPM4 vs p.S1044C**	ns	**	**	**
**WT-TRPM4 vs p.P1204L**	*	***	**	**
**WT-TRPM4 vs p.A101T/P1204L**	ns	**	*	ns

ns = non significant; * P<0.05; ** P<0.005; *** P<0.001
